# Supplementary material for: Sequential Sampling of the Gastrointestinal Tract to Characterize the Entire Digestive Microbiome in Japanese Subjects
Source: Microorganisms. 2024 Jun 28;12(7):1324. doi: 10.3390/microorganisms12071324 (PMC11279317; doi:10.3390/microorganisms12071324)
Supplement: Supplementary file 1 [file microorganisms-12-01324-s001.zip › microorganisms-3079837-supplementary.pdf]

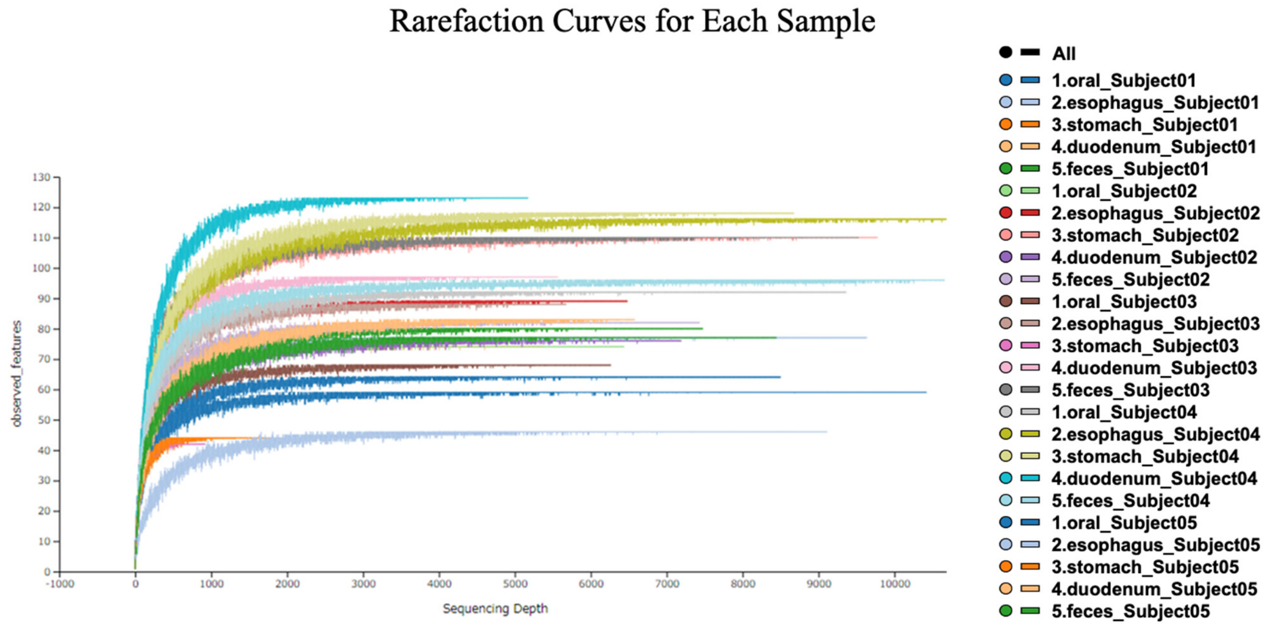

Supplement Figure S1: The rarefaction curves indicate that most samples reached a plateau at approximately 1000 reads, demonstrating that the bacterial richness in these samples was fully captured by the number of sequences analyzed.

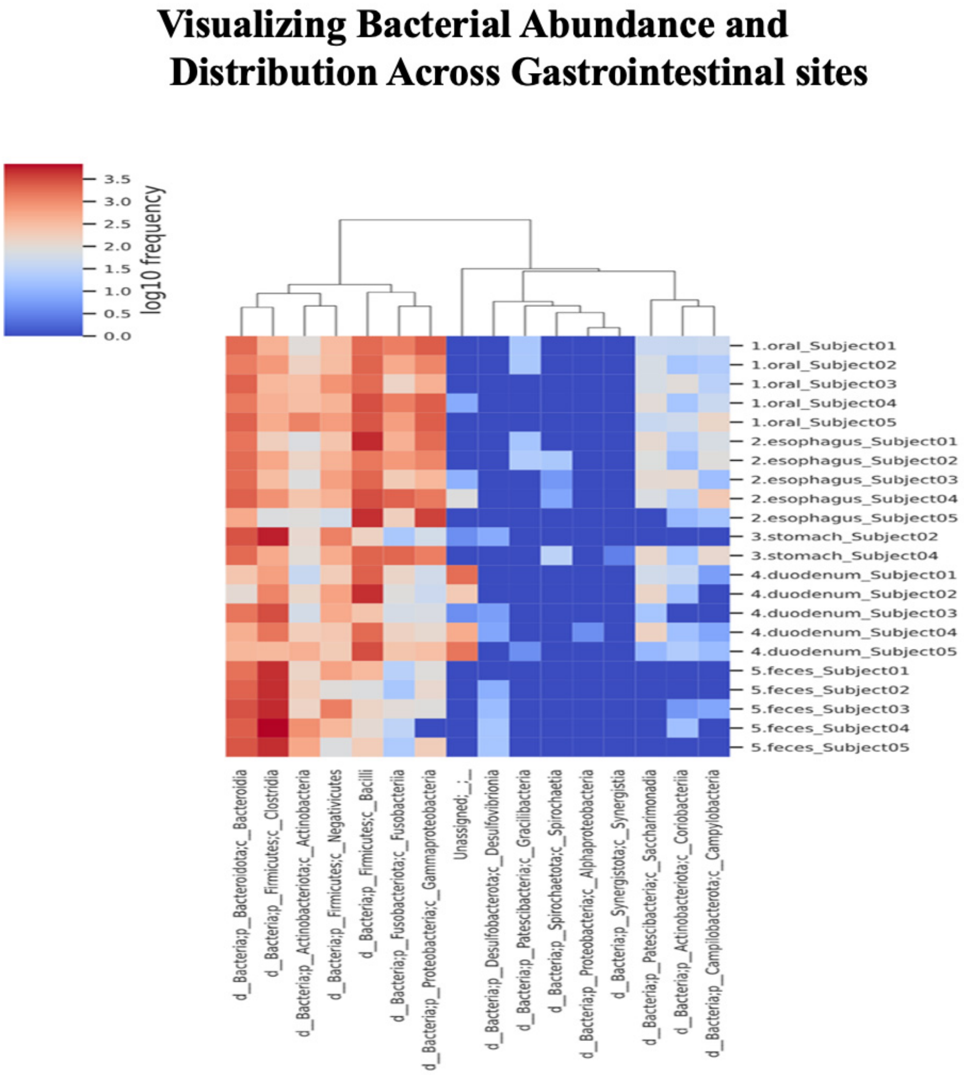

Supplement Figure S2. Visualizing bacterial abundance and distribution across gastrointestinal sites. The heatmap shows the abundance and distribution of bacteria based on 5000 reads. There is no significant difference between 1000 reads and 5000 reads. All subjects meet this criterion except subjects 1, 3 and 4 in the stomach, where similar trends were observed. Darker the red indicates higher abundance, while darker the blue indicates lower abundance. Rows represent samples sorted by collection sites, and columns represent assigned bacterial species. The top dendrogram shows that species with biological similarity cluster together regardless of collection site. In addition, it is clear that the bacterial distribution in fecal samples (bottom five rows) differs from that in the upper gastrointestinal tract, spanning from the oral cavity to the duodenum.

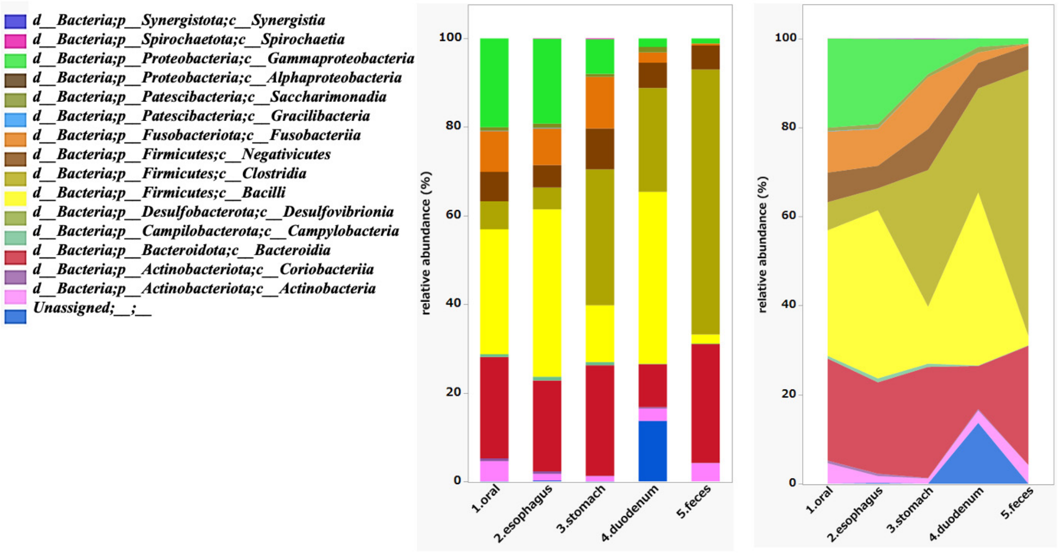

Supplement Figure 3. Class-level microbiome. Class-level microbiome. The charts provided include a stacked bar chart and an area chart analyzed based on 5000 reads. **Stacked Bar Chart**: The left graph illustrates the relative abundance of different bacterial taxa in different gastrointestinal sites, such as the oral cavity, esophagus, stomach, duodenum, and feces. Each color corresponds to a distinct bacterial group, with the height of each colored section representing the proportion of that group in the sample from each site. **Area Chart**: The right graph similarly shows the relative abundance of bacterial taxa, but uses continuous areas to show how the proportions of different bacterial groups change across sites. The distribution of microorganisms varies throughout the GI tract: *Fusobacteria* and *Bacilli* are dominant in the upper GI tract, while *Clostridia* and *Bacteroidia* are dominant in the feces. *Negativicutes* and *Actinobacteria* are present throughout the digestive tract.
